# Assessing the exercise‐related kinetics of circulating cell‐free DNA, circulating tumour DNA, DNase I activity and cytokines in patients with solid tumours: A pilot study

**DOI:** 10.1113/EP092167

**Published:** 2025-03-26

**Authors:** Elmo W. I. Neuberger, Alexandra Brahmer, Tobias Ehlert, Suzan Botzenhardt, Alfonso De Falco, Birgit Enders, Patricia S. Hähnel, Achim Heintz, Carl C. Schimanski, Thomas Kindler, Perikles Simon

**Affiliations:** ^1^ Department of Sports Medicine, Rehabilitation and Disease Prevention Johannes Gutenberg University Mainz Mainz Germany; ^2^ West German Proton Therapy Center Essen (WPE) University Hospital Essen Essen Germany; ^3^ University Cancer Center (UCT), University Medical Center Mainz Mainz Germany; ^4^ Department of Visceral Surgery Marienhaus Klinikum Mainz, Academic Teaching Hospital Mainz Germany; ^5^ 2nd Department of Internal Medicine Municipal Hospital Darmstadt Darmstadt Germany

**Keywords:** cancer, circulating cell‐free DNA, circulating tumour DNA, cytokine, exercise, neutrophil, solid tumour

## Abstract

Circulating cell‐free DNA (cfDNA), circulating tumour DNA (ctDNA) and inflammatory cytokines have prognostic and predictive value in oncology. However, the effects of acute exercise on cfDNA levels are unknown. Here, we explore the kinetics of cfDNA, ctDNA and cytokines upon an incremental exercise test in a pilot cohort of cancer patients compared with healthy control subjects. Patients with solid tumours (*n* = 12) and age‐matched control subjects (*n* = 6) were recruited to perform an all‐out cardiopulmonary bicycle test. Blood samples were collected before (Pre), directly after (Post) and 90 min after the test (+90 min), and the cfDNA, ctDNA (Kirsten rat sarcoma viral oncogene homolog (*KRAS*) mutations), DNase I activity and cytokine levels were measured. Cardiopulmonary exercise testing was easily feasible in cancer patients, and data from eight patients and five control subjects were available for exploratory statistical evaluation. The cfDNA levels increased from Pre to Post and decreased to baseline at +90 min in all subjects. The cfDNA concentrations and DNase I activity were clearly correlated in the control but not in the cancer group. Neutrophil‐associated myeloperoxidase (MPO), calprotectin (MRP 8/14), and lipocalin A (NGAL) showed strong responses to exercise. The percentage of ctDNA, detected in only one cancer patient, decreased after acute exercise. In our study, we could safely perform cardiopulmonary exercise testing with patients with different cancer entities and subsequently run biomarker analyses. Our results hint at an exercise‐triggered release of cfDNA and neutrophil‐derived cytokines in cancer patients.

## Abstract (highlights)

- **What is the central question of this study?**
  How does acute exercise affect the release and clearance of circulating cell‐free DNA in patients with solid tumours in comparison to healthy control subjects?
- **What is the main finding and its importance?**
  Incremental exercise immediately increases the levels of circulating cell‐free DNA and the DNase I activity in patients and control subjects. Healthy subjects show larger increases and decreases, whereas, importantly, in patients the levels normalize after 90 min of rest. Given that exercise can affect the proportion of circulating tumour DNA, our results highlight the importance of considering exercise as a preanalytical factor for cancer liquid biopsies.

## 1 INTRODUCTION

Circulating cell‐free DNA (cfDNA) consists of fragments of genomic DNA, including a tumour‐derived DNA fraction (circulating tumour DNA (ctDNA)), in addition to fragmented or circulatory mitochondrial DNA (cf‐mtDNA). The ctDNA, in particular, has become an important marker implemented in the field of liquid biopsy for cancer management (Cisneros‐Villanueva et al., 2022). During the last three decades, numerous studies have indicated that cancer patients show higher levels of cfDNA than the healthy population (Fleischhacker & Schmidt, 2007). As reviewed by Bronkhorst et al. (2019), cfDNA and ctDNA concentrations are correlated with tumour size, disease stage and metastatic burden. The clearance mechanisms are not clarified in detail; however, at least three mechanisms are described, including direct degradation by nucleases, including DNase I and DNase1L3, active uptake by the reticuloendothelial system in the liver and spleen, and passive filtration by the renal system (reviewed by Han & Lo, 2021).

Multiple screening approaches have been developed to detect tumour‐specific genomic and epigenetic alterations. In addition to PCR, next‐generation sequencing (including whole‐exome or whole‐genome sequencing), cancer personalized profiling by deep sequencing (CAPP‐Seq; Newman et al., 2014), tagged‐amplicon deep sequencing (TAm‐Seq; Forshew et al., 2012) and targeted or whole‐genome methylation sequencing (Liu et al., 2020) were developed to detect mutations, copy number aberrations, cfDNA fragmentation profiles (Mouliere et al., 2018) or cancer‐specific epigenetic signatures (Liu et al., 2020). Detection of Kirsten rat sarcoma viral oncogene homolog (*KRAS*) point mutations is specifically important because anti‐EGFR therapy is ineffective in the presence of *KRAS* mutations (Lièvre et al., 2006). We developed a highly specific nested qPCR targeting *KRAS* point mutations to detect and quantify minute amounts of circulating ctDNA, which was used to monitor the release of ctDNA during surgery (Ehlert et al., 2017).

Although the applications of cfDNA in cancer liquid biopsy are increasing, the significance of preanalytics and standardization has become increasingly visible. The ctDNA is very low in concentration in relationship to background cfDNA, making it hard to detect. The choice of anticoagulant, blood processing and cfDNA isolation method strongly determine the composition of cfDNA populations in a sample (Ungerer et al., 2020). Especially for, but not limited to, the early‐stage diagnosis of cancers, where ctDNA concentrations are very low, it is mandatory not to dilute ctDNA by increasing background cfDNA (Bronkhorst et al., 2019). In this context, it is important to understand to what extent environmental factors, such as physical exercise, circadian rhythms or nutrition, might influence the outcome of cancer liquid biopsies and therefore should be considered for the standardization of sampling for liquid biopsies (Bronkhorst et al., 2019; Kuligina et al., 2021; Ungerer et al., 2020).

Physical exercise has shifted from a perceived threat to patients to a widely accepted supportive treatment in cancer therapy and rehabilitation in only a few decades. Physical exercise not only reduces the incidence of cancer but also reduces cancer recurrences for some types of cancers and inhibits tumour growth (Hojman et al., 2018). It is assumed that the positive effects of exercise are partly related to the dynamic interaction between the tumour microenvironment and host immune reactivity, mediated by cytokines and other signalling molecules (Kartikasari et al., 2021). The feasibility and safety of exercise interventions in cancer patient cohorts of nearly all exercise intensities have been demonstrated in various patient studies (e.g., An et al., 2021; Heywood et al., 2017; Kartikasari et al., 2021). Even ‘exotic’ and very strenuous disciplines, such as high‐intensity interval training (Mijwel et al., 2020) or high‐intensity strength training (De Backer et al., 2007), find use. Intriguingly, acute exercise bouts lead to an immediate rise in cfDNA levels in healthy conditions and in several pathologies (Breitbach et al., 2014; Neuberger et al., 2021; Tug et al., 2015). The kinetics of cfDNA in response to incremental exercise testing in patients with solid tumour is unknown.

Here, we elucidate the feasibility of assessing the impact of cardiopulmonary exercise testing on circulating DNA species in a small cohort of patients with heterogeneous cancer entities. Via minimally invasive direct measurement of cfDNA, we examined the effect of an acute physical exercise bout on cfDNA levels in cancer patients. Moreover, using highly specific nested quantitative PCR (qPCR) targeting *KRAS* point mutations (ctDNA) we detected one case of *KRAS* point mutation (ctDNA) in this exercise setting. Additionally, we analysed the kinetics of inflammatory cytokines in a subset of randomly selected samples.

## 2 MATERIALS AND METHODS

### 2.1 Ethical approval and consent to participate

**FIGURE 1:**
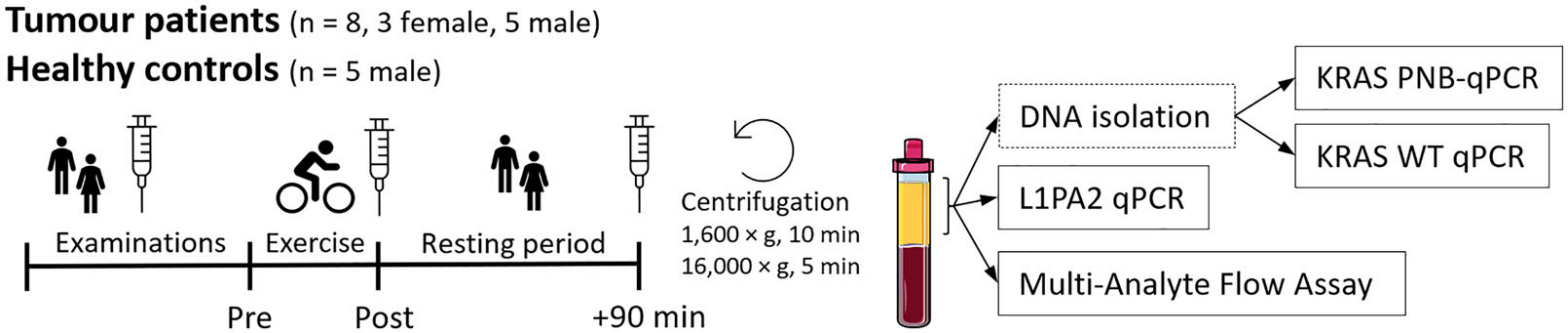
Study design. K_3_‐EDTA plasma was collected before, directly after and 90 min after all‐out incremental cycling exercise and centrifuged twice before cfDNA, *KRAS* ctDNA and circulatory cytokine analyses. Abbreviations: cfDNA, cell‐free DNA; ctDNA, circulating tumour DNA; L1PA2, long interpersed element type 1 subfamily PA2; *KRAS*, Kirsten rat sarcoma viral oncogene homolog; WT, wild‐type; qPCR, quantitative PCR; PNBqPCR, pooled nested WT blocking qPCR.

The study was conducted according to the guidelines of the *Declaration of Helsinki* and approved by the Ethics Commission of the state of Rhineland‐Palatinate, Mainz, Germany [837.033.11 (7575)]. Written informed consent was obtained from all study participants. The protocol is illustrated schematically in Figure 1.

### 2.2 Participants and sample collection

A total of 12 tumour patients and six age‐matched healthy subjects were recruited at the University Medical Center Mainz, Mainz, Germany, and the St. Hildegardis Hospital Mainz, Mainz, Germany, and the Department of Sports Medicine of the Johannes Gutenberg‐University of Mainz, Mainz, Germany. Inclusion criteria for the tumour group were a located solid tumour, and age between 40 and 70 years. Exclusion criteria were as follows: chemotherapy within the last 48 h before the examination; radiation within the last 7 days before the examination; enterostomy; untreated anaemia; and indications of diseases of the lungs, cardiovascular system, liver, kidney or seizure disorders that rule out acute exercise. Furthermore, known addictions (alcohol or drugs) and the use of anticoagulant medication impeded inclusion in the study. Before the exercise test, the sports capability of all the participants was confirmed by a physician at the Department of Sports Medicine. All exercise tests and blood sampling were conducted at the Department of Sports Medicine, Mainz, Germany.

### 2.3 Cardiopulmonary exercise test

To determine the peak oxygen uptake (${\dot{V}}_{O_{2}\mathrm{peak}}$), a cardiopulmonary exercise test was performed, supervised by an experienced exercise physiologist. The stepwise incremental cycling exercise test until exhaustion was performed on an ergoselect 200 Cycling ergometer (ergoline GmbH, Bitz, Germany). Respiratory gas exchange data were recorded breath by breath by a metabolic unit (Ergostik, Geratherm Respiratory GmbH, Bad Kissingen, Germany), and the ECG was recorded continuously using Cardiopart 12 Blue ECG Pro Amedtec (AMEDTEC Medizintechnik Aue GmbH, Aue, Germany). The individualized workload protocol contained steps of 3 min duration with different initial loads and increasing resistance. An experienced physician individualized the protocol based on the treatment history, comorbidities and physical condition. Blood pressure was monitored throughout rest and exercise as recommended (American Thoracic Society & American College of Chest Physicians, 2003). At the end of each workload, the subjects were asked for their rating of perceived exhaustion (RPE) (Williams, 2017). Lactate concentrations in capillary blood taken from the earlobe after each step of the incremental test were determined with an automatic lactate analyser Biosen 5130 (EKF Diagnostics, Magdeburg, Germany). The exercise test was terminated when subjective exhaustion was reached or if any of the general indications for exercise termination were observed (American Thoracic Society & American College of Chest Physicians, 2003). Notably, the cardiopulmonary capacity was determined at peak exercise (${\dot{V}}_{O_{2}\mathrm{peak}}$), as recommended for clinical populations, and not at maximum oxygen uptake (American Thoracic Society & American College of Chest Physicians, 2003).

### 2.4 Blood handling, processing and DNA isolation

Venous blood samples were taken before the test, directly after and +90 min after the end of the test using K_3_‐EDTA Monovettes (Sarstedt, Nümbrecht, Germany). All blood samples were centrifuged for 10 min at 1600*g* within 10 min after withdrawal, and the resulting plasma was centrifuged again in a new tube for another 5 min at 16 000*g*, both at 4°C. Plasma samples were stored at −20°C. cfDNA was isolated from fractions of the plasma samples using the QIAamp Circulating Nucleic Acid Kit (Qiagen, Hilden, Germany) following the manufacturer's instructions with two changes. First, no carrier RNA was used in the protocol, and second, the DNA was eluted in water instead of the included buffer.

### 2.5 Quantification of cfDNA and ctDNA

The concentrations of cfDNA and ctDNA were measured using three different qPCR approaches. First, total cfDNA concentrations were measured using an ultrasensitive qPCR approach amplifying genomic DNA in diluted plasma, without the need for DNA isolation. Second, isolated DNA was used to quantify the seven most common *KRAS*‐specific mutations, and third, mutation‐independent *KRAS* qPCR was used to determine the percentage of mutated fragments.

Total genomic cfDNA was quantified from diluted plasma by amplifying an L1PA2 retrotransposable element with ∼3340 hits in the human genome, as described previously (Breitbach et al., 2014). In short, the plasma was diluted 1:40 with PCR‐grade water, then mixed with Tego Buffer, Velocity Polymerase, MgCl_2_ (all from Bioline, Luckenwalde, Germany), SYBR Green, FITC (both from Sigma‐Aldrich Co., Taufkirchen, Germany) and 0.34 mM of a primer pair specific for a sequence of 90 bp in the aforementioned LINE element (5′‐TGCCGCAATAAACATACGTG‐3′ and 5′‐GACCCAGCCATCCCATTAC‐3′). A CFX384 Bio‐Rad (Bio‐Rad, Munich, Germany) cycler was used with the following protocol: 35 cycles of 94°C for 10 s, 64°C for 40 s and 75°C for 10 s, followed by a melting curve. All measurements were run in triplicate.

cfDNA bearing cancer‐specific *KRAS* mutations (ctDNA) was amplified with a highly sensitive nested qPCR approach called PNB‐qPCR (pooled, nested WT‐blocking qPCR) (Ehlert et al., 2017). With this technique, we are able to quantify down to 0.01% and to detect down to 0.003% of mutant DNA of the seven most common *KRAS* mutations, namely G12D, G12V, G12A, G12S, G12C, G12R and G13D (Ehlert et al., 2017). The nested qPCR protocol comprised a first‐ and a second‐round PCR. In the first round of PCR, a 110 bp PCR product was amplified, including a wild‐type (WT) specific 3'C6 amine blocking primer for mutation enrichment (for primer sequences, see supplementary material in the paper by Ehlert et al., 2017). The PCR protocol consisted of 20 cycles of 10 s at 98°C and 30 s at 69°C, followed by 5 min of final elongation at 72°C, including 0.002 U/µL Phusion® Hot Start Flex DNA Polymerase with the accompanying 1× Phusion® HF buffer, 0.5 mM MgCl_2_ (New England Biolabs, Ipswich, MA, USA), 200 µM dNTPs, 400 µM outer primer mix, 1 µM blocking primer, and 14 µL of template and H_2_O in a final volume of 50 µL. In the second round of PCR, the pre‐amplified sample (1:50 dilution) was amplified with seven mutation‐specific amplification‐refractory mutation system (ARMS) primers, including a locked nucleic acid (LNA) probe for maximum specificity. The reaction mix included 1× SsoAdvanced™ Universal Probes Supermix (Bio‐Rad, Munich, Germany), 400 nM forward ARMS and reverse primers, 200 nM LNA probe, and 3.2 µL of template for a total of 8 µL per well. There were three different qPCR protocols, depending on the primer pair used.

For the mutation‐independent quantification of *KRAS*, exon 2 was targeted outside of the mutation hotspots (5′‐GAATATAAACTTGTGGTAGTTGGAGC‐3′ and 5′‐ CTGAATTAGCTGTATCGTCAAGG‐3′) using the *KRAS* WT LNA probe ([6FAM]CTC[+T][+T]GC[+C][+T]ACGC[+C]A[BHQ1]). The reaction mix of this qPCR was identical to the second round of the PNB‐qPCR. The qPCR conditions were 2 min at 95°C followed by 50 cycles of 5 s at 95°C and 30 s at 67°C, and a final elongation step of 5 min at 72°C.

### 2.6 DNase activity ELISA

An ELISA to measure DNase I activity was performed with 9 µL of plasma for all plasma samples according to the manufacturer's instructions (ORGENTEC, Mainz, Germany).

### 2.7 Multi‐analyte flow assay

Cytokines were quantified using a human inflammation panel (13‐plex) and a human vascular inflammation panel (12‐plex) (LEGENDplex™, Biolegend) according to the manufacturer's instructions. Diluted plasma samples were mixed with bead‐coated capture antibodies specific for interleukin (IL)‐1β, interferon‐α2 (INF‐α2), interferon‐γ (INF‐γ), tumour necrosis factor‐α (TNF‐α), monocyte chemoattractant protein‐1 (MCP‐1), IL‐6, IL‐8, IL‐10, IL‐12p70, IL‐17A, IL‐18, IL‐23, IL‐33 (inflammation panel) or myoglobin, calprotectin (MRP8/14), neutrophil gelatinase‐associated lipocalin (NGAL), matrix metalloproteinase‐2 (MMP‐2), osteopontin (OPN), myeloperoxidase (MPO), serum amyloid A (SAA), insulin‐like growth factor binding protein 4 (IGFBP‐4), intercellular adhesion molecule 1 (ICAM‐1), vascular cell adhesion molecule 1 (VCAM‐1), matrix metalloproteinase 9 (MMP‐9) and cystatin C (vascular inflammation). After an incubation time of 1 h, the beads were washed twice before adding the biotin‐labelled detection antibodies for 1 h. Following a final incubation with streptavidin‐PE, the beads were analysed using a FACSCanto II cytometer (BD). Analysis was performed using the LEGENDplex™ analysis software v.8.0. The software distinguishes between 12 or 13 different analytes based on bead size and internal dye.

### 2.8 Statistical analysis

All statistical tests were performed using R (v.4.1.0) and the rstatix package (v.0.7.2). The figures were created using ggplot2 (v.3.1.4). Normal distribution of the data was tested with the Shapiro‒Wilk test. The homogeneity of variances was assessed with Levene's test. The cfDNA and cytokine data were log normalized to achieve a normal distribution. For the graphical presentation of the data, the back‐transformed mean of the log values is displayed. In the text, the means and SD of the untransformed data are presented. If not otherwise stated, parametric mixed ANOVA tests with Bonferroni–Holm adjusted *post hoc* tests were computed to identify statistically significant differences. Student's paired or unpaired *t*‐tests or Wilcoxon tests were used for normal or non‐normally distributed data, respectively. Correlations for the normally distributed data were computed using the Pearson correlation coefficient. A *p*‐value ≤ 0.05 was considered significant.

## 3 RESULTS

### 3.1 Study cohort

Twelve tumour patients and six healthy control subjects were recruited for inclusion in the study. One patient was excluded prior to the exercise test because of high blood pressure on two separate occasions. One test could not be performed owing to technical difficulties, and the patient abandoned the study afterwards. Two other patients completed the test, but no blood could be taken directly after the exercise test; therefore, no data were available for analysis. Additionally, the 90 min resting sample could not be taken from one patient because of insufficient blood flow. This patient was not excluded from the study. For one control patient, ${\dot{V}}_{O_{2}\mathrm{peak}}$ and RER could not be determined owing to unreliable data. Therefore, eight tumour patients (five male and three female) and five age‐matched healthy male control subjects were included for exploratory statistical evaluation. In the patient cohort, four subjects had colon cancer, two of whom had metastases. Two patients had oesophageal carcinoma, and one patient had gastric cancer. One had mammary and thyroid carcinoma with distant metastases.

### 3.2 Bicycle ergometry and physiological parameters

The starting resistance for the stepwise bicycle ergometry test ranged from 20 to 75 W with a mean of 26.3 ± 5.8 W for the tumour group and 58 ± 16.0 W for the control group. The starting watts were chosen with respect to the estimated fitness of the subjects. The number of completed steps was not significantly different between the tumour group (4.2 ± 1.5) and the control group (6.3 ± 2.1), *p* = 0.107 (Table 1). However, the control group reached significantly higher maximal watts (*p* = 0.007). Thus, the control group performed higher loads for a longer time. In line with this, the control subjects showed a significantly higher lactate increase at exhaustion, with a mean of 9.9 ± 3.4 mmol/L compared with the control subjects (5.7 ± 1.8 mmol/L), and a significantly higher mean ${\dot{V}}_{O_{2}\mathrm{peak}}$ (35.3 ± 6.3 mL/min/kg) compared with the tumour group (22.5 ± 9.3 mL/min/kg). However, the RPE and RER were similar between the groups (Table 1), indicating that all subjects reached the level of maximum exhaustion (*p *> 0.05).

**TABLE 1:** Baseline and exercise performance characteristics of study participants allocated by group (T = Tumour, C = Control) and time point (Pre or Post exercise).

Characteristic	Time point Pre	*p*‐value between condition at time point Pre	Time point Post	*p*‐value within condition (Pre to Post)	*p*‐value between condition at time point Post
*n* (male/female)	T: 8 (5/3) C: 5 (5/0)				
Age, years	T: 53.5 ± 3.2 C: 54.8 ± 6.6	*p*_T _= 0.864			
BMI, kg/m^2^	T: 25.6 ± 3.2 C: 24.6 ± 3.4	*p*_T_ = 0.607			
Heart rate, /min	T: 92.1 ± 12.2 C: 72.6 ± 9.1	***p*_T_ = 0.011**	T: 151.7 ± 24.6 C: 177 ± 15.6	**T: *p*_T _< 0.001** **C: *p*_T _< 0.001**	*p*_T =_ 0.067
Systolic BP, mmHg	T: 115.3 ± 14.0 C: 129.5 ± 14.8	*p*_T _= 0.147	T: 180.4 ± 50.2 † C: 222.4 ± 29.7	**T: *p*_T _= 0.006** **C: *p*_T _< 0.001**	*p*_T _= 0.128
Diastolic BP, mmHg	T: 77.3 ± 9.2 C: 83.0 ± 15.4	*p*_W_ = 0.152	T: 86.8 ± 20.4 C: 93.6 ± 10.3	T: *p*_W _= 0.280 C: *p*_T_≤ 0.537	*p*_T _= 0.516
Lactate, mmol/L	T: 1.1 ± 0.28 C: 0.9 ± 0.15	*p*_T _= 0.089	T: 5.7 ± 1.8 C: 9.9 ± 3.4	**T: *p*_T _< 0.001** **C: *P*_T _< 0.001**	***p*_T _= 0.017 **
O_2_ uptake, ml/min/kg	T: 5.0 ± 1.1 C: 4.5 ± 0.2	*p*_T_ = 0.301	T: 22.5 ± 9.3 C: 35,3 ± 6.4	**T: *p*_W _< 0.001** **C: *p*_T _< 0.001**	***p*_W _= 0.030**
RER			T: 1.13 ± 0.09 C: 1.1 ± 0.06		*p*_T _= 0.52
RPE			T: 18.4 ± 1.5 C: 18.6 ± 1.1		*p*_T _= 0.781
Steps until exhaustion			T: 4.2 ± 1.5 C: 6.3 ± 2.1		*p*_W_ = 0.107
Maximal watts			T: 122.5 ± 54.4 C: 227.0 ± 57.2		***p*_T _= 0.007**

*Note*: Data are presented as the mean ± SD. The *p*‐values for between‐group differences were calculated using Student's unpaired *t*‐tests (*p*_T_) or Wilcoxon tests (*p*_W_). The Pre to Post exercise within‐group differences were calculated using Student's paired *t*‐tests (*p*_T_) or Wilcoxon tests (*p*_W_), depending on normal distribution. The tumour group included patients with colon cancer (*n* = 2), metastatic colon cancer (*n* = 2), oesophageal cancer (*n* = 2), gastric cancer (*n* = 1) and mammary and thyroid carcinoma with distant metastases (*n* = 1).

Abbreviations: BMI, body mass index; BP, blood pressure; RER, respiratory exchange ratio; RPE, rating of perceived exertion.

### 3.3 cfDNA and DNase I activity analysis

**FIGURE 2:**
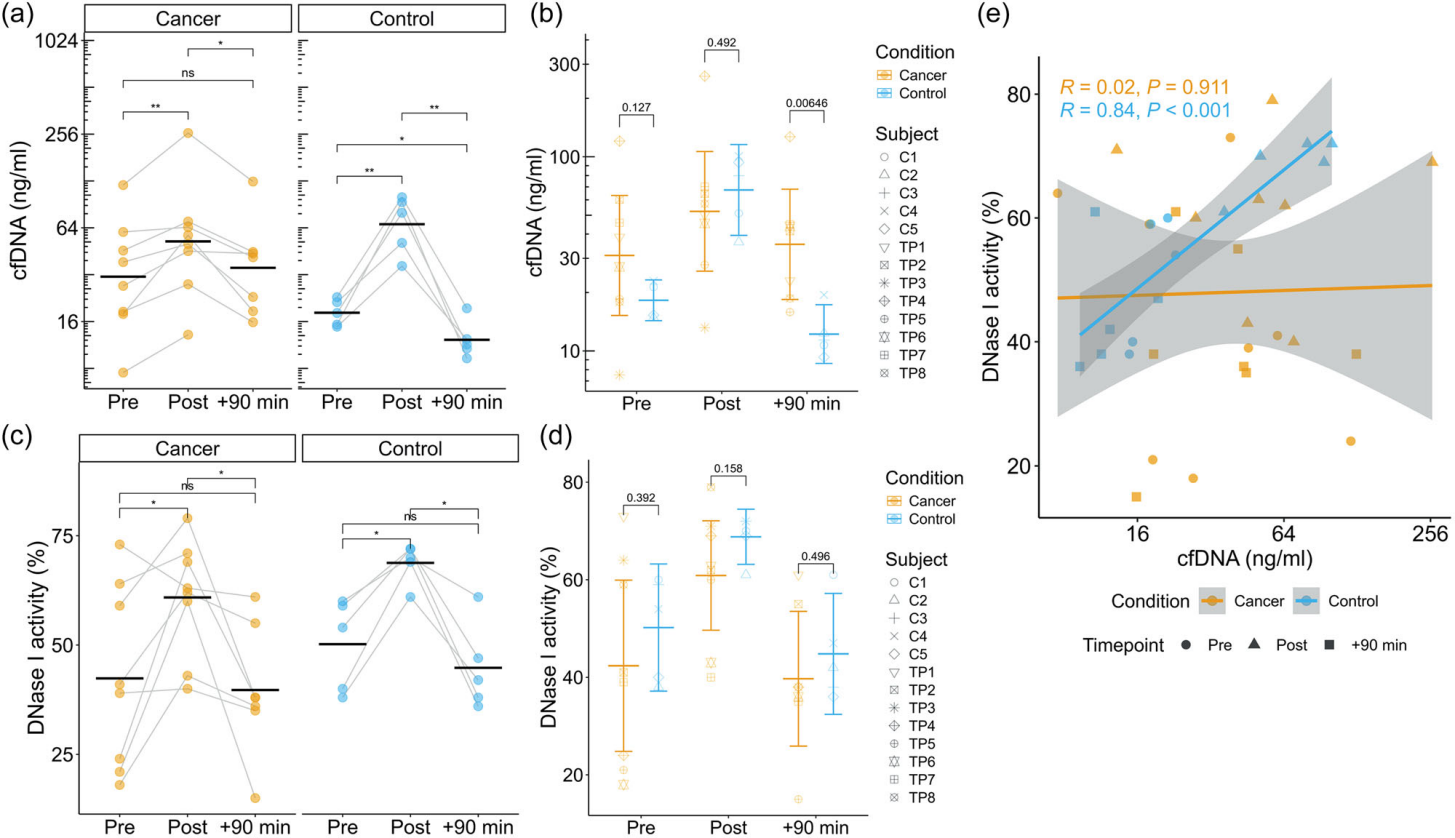
(a) Individual development of cfDNA concentrations in cancer and control groups over test time. (b) Comparison of cfDNA concentrations in the tumour and control groups at different time points. The mean and 95% confidence interval values are shown. (c, d) DNase I activity (as a percentage) in tumour and control groups over test time (c) and between groups (d). (e) Correlation between cfDNA concentration and DNase I activity (as a percentage). ^*^ *p* < 0.05; ^**^ *p* < 0.01; ns, not significant. Abbreviation: cfDNA, cell‐free DNA.

Initially, we wanted to examine the release and clearance kinetics of total cfDNA in response to an all‐out physical exercise test in the tumour group and the control group. A mixed ANOVA on the cfDNA concentrations revealed a statistically significant interaction between the time points Pre, Post, +90 min and the condition cancer versus healthy [*F*(2, 20) = 15.348, *p *< 0.001, partial η^2^ = 0.605], indicating that the cfDNA concentrations of the two groups differed over the time course of the bicycle test. As shown in Figure 2a, cfDNA increased significantly, 1.75‐fold, in the cancer group, from 42.0 to 73.6 ng/mL, and decreased to 44.9 ng/mL at +90 min. In the control subjects, the cfDNA concentration increased 3.90‐fold, from 18.5 to 72.3 ng/mL, then decreased to 12.6 ng/mL. The fold‐change increases (Post/Pre) and decreases (Post/+90 min) were significantly higher in the control group than in the cancer group (increase 3.90 vs. 1.75, *p* = 0.008 and decrease 6.0 vs. 1.89, *p* = 0.002, respectively). As indicated in Figure 2b, no significant difference was detected between groups at the time points Pre (41.99 ± 35.97 versus 18.5 ± 3.59 ng/mL, *p* = 0.127) and Post (73.62 ± 77.71 versus 72.32 ± 27.61 ng/mL, *p* = 0.492), but the time point +90 min was significant (*P* = 0.006), with 44.90 ± 38.19 ng/mL in the cancer group compared with 12.63 ± 3.97 ng/mL in the control group.

The kinetics of DNase I activity were more consistent in the control group than in the tumour group, and all subjects showed increases in response to acute exercise. DNase I activity (as a percentage) increased significantly from Pre (50.2 ± 10.5) to Post (68.8 ± 4.5, *p* = 0.01) and decreased to 44.8 ± 9.9 at +90 min (Figure 2d). In the cancer group, DNase I activity increased from 42.4 ± 21.0 to 60.9 ± 13.4 (*p* = 0.046) and decreased to 39.7 ± 14.9. In both groups, no differences were detected from Pre to +90 min (Figure 2c). Additionally, no significant differences were found between the groups at different time points (Figure 2d).

Importantly, there was a clear correlation between cfDNA concentration and DNase I activity in the control group (Figure 2e), with a Pearson correlation coefficient of *R *= 0.84, *p* < 0.01, whereas the parameters were not correlated in the cancer group.

### 3.4 ctDNA

**FIGURE 3:**
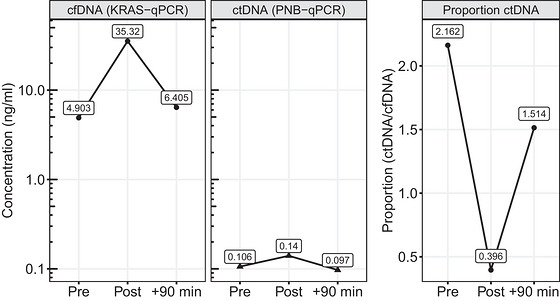
Kinetics of cfDNA, *KRAS* ctDNA, and the proportion of ctDNA (ctDNA/cfDNA) in the single subject who tested positive for the circulating *KRAS* mutation. Abbreviations: cfDNA, cell‐free DNA; *KRAS*, Kirsten rat sarcoma viral oncogene; ctDNA, circulating tumour DNA; PNB‐qPCR, pooled nested wild‐type blocking quantitative PCR.

Next, we screened the plasma samples from all cancer patients taken during the physical exercise test for circulating DNA containing *KRAS* mutations (ctDNA) by PNB‐qPCR. One patient was *KRAS* mutation positive, and mutated fragments were quantified in all three samples taken. ctDNA concentrations were 0.106 ng/mL before the test (Pre), 0.14 ng/mL directly after the test (Post), and 0.097 ng/mL at +90 min. As a percentage of total cfDNA, this equals 2.16%, 0.396% and 1.51% of total cfDNA, respectively. The ctDNA percentages were calculated as a quotient of the concentration of the mutated *KRAS* sequence and total *KRAS* DNA concentration in the isolated cfDNA quantified by qPCR (Figure 3).

### 3.5 Multi‐analyte flow assay

**FIGURE 4:**
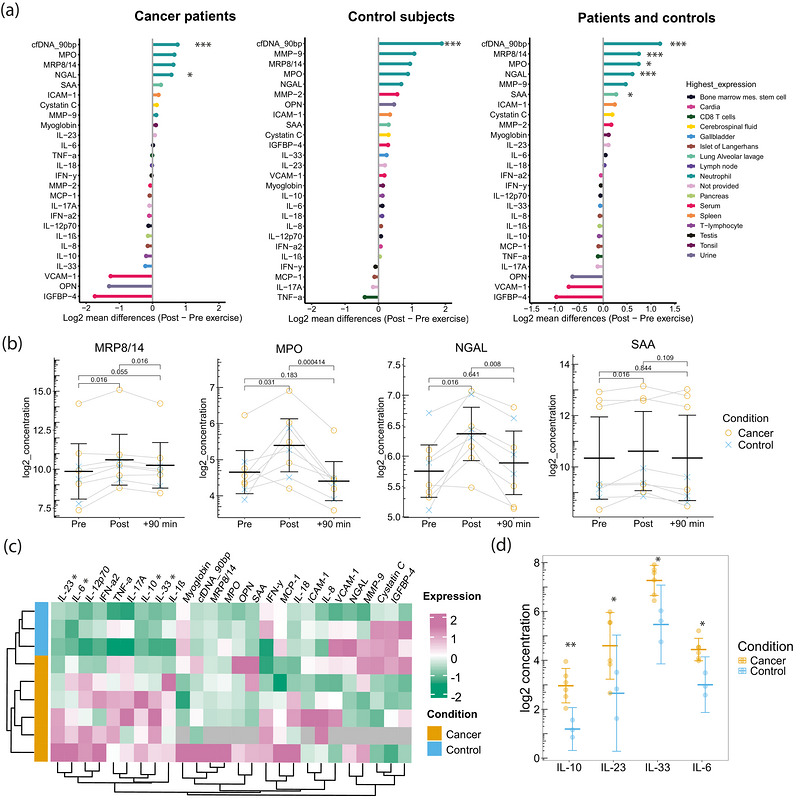
Circulatory cytokine expression changes in response to exercise. (a) Log_2_ mean differences from Pre to Post exercise in cancer patients, control subjects and pooled samples (cancer and control subjects) indicate that neutrophils mainly contribute to changes in expression. The highest expressions for the proteins were taken from Gene–cards—Human Genes | Gene Database | Gene Search providing the highest protein expression in normal tissues and cell lines. (b) Kinetics of the differentially expressed plasma proteins. (c) Heatmap for *z*‐normalized expression at baseline levels in cancer patients and healthy control subjects. Grey fields display values below the limit of detection. (d) Exploratory description of significantly elevated cytokines in cancer patients compared with healthy control subjects at baseline. Student's paired or unpaired *t*‐tests or Wilcoxon tests were conducted to compare the groups, without *p*‐value correction (^*^ *p* < 0.05; ^**^ *p* < 0.01; ^***^ *p* < 0.001; ns, not significant). Abbreviations: cfDNA, cell‐free DNA; MPO, myeloperoxidase; MRP8/14, calprotectin; NGAL, neutrophil gelatinase‐associated lipocalin; SAA, serum amyloid‐A; ICAM, intracellular adhesion molecule; MMP, matrix metalloproteinase; IL, interleukin; TNF, tumour necrosis factor; IFN, interferone; MCP, monocyte chemoattractant protein; VCAM, vascular cell adhesion molecule; OPN, osteopontin; IGFBP, insulin‐like growth factor‐binding protein.

In a subset of randomly selected samples including six cancer patients and three healthy controls, we studied the kinetics of 25 inflammatory proteins included in the LEGENDplex™ inflammation panel (13‐plex) and a human vascular inflammation panel (12‐plex). We could show that meaningful measurements are possible for cancer patients in the selected exercise setting. As shown in Figure 4a, the concentration of circulatory proteins increased or decreased in response to acute exercise in the control group and in cancer patients. MPO, MRP8/14, NGAL and SAA showed the strongest log_2_ mean differences (Post vs. Pre exercise). IGFBP‐4, OPN, and VCAM‐1 were the most strongly downregulated proteins. The picture was similar in the cancer patients and control subjects. A combination of the data (patients and control subjects) showed that MRP8/14, MPO, NGAL and SAA were significantly upregulated (*P* < 0.05; Figure 4a). None of the cytokines was significantly regulated from Pre exercise to +90 min. Figure 4b illustrates the kinetics of the four significantly upregulated cytokines.

A cluster analysis for the *z*‐transformed protein concentration values shows a differentiation into the control and cancer groups (Figure 4c). Student's unpaired *t* test comparisons of the normally distributed data with homogeneous variances indicated that IL‐10 (*P* = 0.001), IL‐6 (*P* = 0.011), IL‐33 (*P* = 0.018) and IL‐23 (*P* = 0.048) differed significantly at the time point Pre between the cancer and control groups (Figure 4d). However, owing to the small sample size, the accuracy of the statistical tests is limited.

## 4 DISCUSSION

The purpose of the study was to obtain the first evidence on the effects of acute exercise on the kinetics of cfDNA, ctDNA and DNase I activity in a small pilot cohort of patients with solid cancers in comparison to age‐matched control subjects, in order to be able to plan fully powered follow‐up studies. Cardiopulmonary exercise testing was easily feasible in the tumour group and the healthy control group, and the dropout rate owing to medical, personal or technical reasons was comparable to other studies. In our pilot analysis of blood markers, we found significant increases in cfDNA, DNase I activity and inflammatory cytokines in response to exercise in both groups. The cfDNA kinetics differed significantly between the groups. A clear correlation between cfDNA and DNase I activity could be detected in healthy subjects but not in cancer patients, indicating disordered DNase I and cfDNA homeostasis. Neutrophil‐associated inflammatory cytokines (MPO, MRP8/14 and NGAL) showed large increases in response to exercise, suggesting a large contribution of neutrophils to the exercise‐mediated effect. In our cancer cohort, we identified one subject who was positive for *KRAS* ctDNA. In this case, the kinetics of cfDNA and ctDNA indicate that exercise does not increase the absolute ctDNA concentration but decreases the percentage of ctDNA compared with the total amount of cfDNA, indicating that exercise can be a relevant preanalytical factor impairing the results of liquid biopsies.

cfDNA is known to circulate in the blood of healthy individuals at low concentrations. Various pathophysiological conditions, including haematopoietic and solid cancers, are associated with increased cfDNA levels. Emerging evidence suggests that cfDNA, in the form of nucleosomes, is not only a waste product of cell death but also has pathogenic or even functional roles (Han & Lo, 2021), including acting as a pro‐inflammatory stimulant and promotor of tumour growth (reviewed by Han & Lo, 2021; Hedrick & Malanchi, 2022). Therefore, low levels of cfDNA at rest and efficient cfDNA clearing during exercise are likely to have beneficial effects. In our age‐matched cohorts, the cancer patients showed ∼2.3‐fold higher cfDNA values at rest compared with healthy subjects (41.99 ± 35.97 vs. 18.5 ± 3.59 ng/mL). Likewise, Mattox et al. (2023) identified ∼2.9‐fold higher levels of cfDNA in a large cohort of patients with pancreatic, colorectal, lung or ovarian cancer in comparison to healthy control subjects (18.1 ± 8.6 vs. 4.3 ± 2.6 ng/mL). Notably, the different absolute cfDNA concentrations might result from preanalytical factors including the DNA isolation method, which significantly affects cfDNA yield (Ungerer et al., 2020). In line with former studies, we found that cfDNA levels increase in response to acute exercise, with more pronounced increases in healthy cohorts than in diseased subjects (Neuberger et al., 2021; Tug et al., 2015). The cfDNA levels increased 1.75‐ or 3.90‐fold in patients and control subjects, respectively. Importantly, this indicates that cancer patients do not have an overshooting cfDNA increase in response to acute exercise.

The clearance of cfDNA is related to the activity of different nucleases (Han & Lo, 2021). As shown by Velders et al. (2014), exercise is a potent stimulus to enhance the activity of circulating DNase I in healthy subjects in response to a stepwise incremental exercise test on a rowing ergometer. We showed that acute exercise triggers DNase I activity in healthy subjects and cancer patients. In both cohorts, the activity increased significantly, reducing to baseline levels at +90 min (Figure 2c). We did not find significant differences in DNase I activity between the groups at any time point analysed (Figure 2d); however, a clear correlation between cfDNA and DNase I activity was detected for healthy control subjects but not for the cancer group (Figure 2e). This finding might indicate a disordered balance between cfDNA and DNase I activity in tumour patients. However, given that the tumour patients had a more or less efficient clearance of the cfDNA, it can be expected that other nucleases contribute to cfDNA homeostasis and that DNase I activity is not the main factor regulating cfDNA levels. Future studies should consider analysing the activity of other relevant nucleases, including DNase1L3. Given that DNase I activity has been described as being up‐ or downregulated in different types of cancer (reviewed by Han & Lo, 2021), larger studies with more homogeneous cohorts could help to identify how and which nuclease counterbalances the increased levels of cfDNA. Notably, Ondracek et al. (2022) studied the effect of 8 months of consequent exercise on DNase I activity in patients with a cardiovascular risk factor. They showed that in the group of subjects who achieved performance gains, the cfDNA levels decreased, whereas DNase activity increased. Moreover, the results from animal studies indicate that DNase I injection led to increased survival time, a reduced number of cancer cells, and inhibition of metastasis (reviewed by Hawes et al., 2015). Thus, long‐term exercise interventions that affect DNase activity could have beneficial effects in cancer patients.

Cytokines have a crucial role in cancer development and serve as the means of communication between the immune system and healthy and malignant cells. Here, we ran pilot tests on the cytokine response after an acute bout of cycling exercise in a subgroup of our cancer patients. We found the highest increases in inflammatory cytokines that are most highly expressed in neutrophils, indicating that neutrophils greatly contribute to the exercise‐mediated effect. Acute physical exercise leads to profound and transient changes in blood cell count and functional capacity of the cells (Campbell & Turner, 2018). This includes the release of cytokines that interact in a coordinated system activating immunospecific pathways and interact with the (host) tissue–tumour microenvironment (Koelwyn et al., 2020). In our study, four cytokines were significantly upregulated in response to acute exercise (Figure 4a, b). None of the cytokines showed significant differences from Pre to +90 min. MPO, MRP8/14 and NGAL were most strongly increased in response to exercise. All of them are highly expressed in neutrophils (Urban et al., 2009). In response to their activation, the cells can extrude neutrophil extracellular traps (NETs). NETs are net‐like structures composed of DNA–histone complexes and proteins, including MPO, NGAL, MRP8/14 and others (Urban et al., 2009). The process of NET release typically takes several hours; however, rapid release mechanisms, called vital netosis, have been described (Jorch & Kubes, 2017; Pilsczek et al., 2010). Using targeted bisulphite sequencing, we have recently shown that in healthy persons, neutrophils are the major source of cfDNA released in response to exercise (Neuberger et al., 2022). More recently, Mattox et al. (2023) identified that neutrophils are the major source of cfDNA in patients with pancreatic, colorectal, lung or ovarian cancer, who show elevated cfDNA levels. Neutrophils have attracted attention because of their role in cancer promotion (Hedrick & Malanchi, 2022; Xiong et al., 2021). The regular activation and renewal of the pool of circulating neutrophils by exercise might have beneficial effects in cancer patients and in the healthy population. Neutrophil‐released cytokines, including neutrophil elastase, which are released in response to exercise, could have beneficial effects on the development of cancer and tumour growth. Cui et al. (2021) showed that neutrophils release catalytical active neutrophil elastase, which kills cancer cells and attenuates tumorigenesis. Next to the effect of acute exercise, we compared the cytokine levels in resting conditions. The plasma levels of IL‐23, IL‐33, IL‐6 and IL‐10 were higher in cancer patients than in healthy control subjects, which has been described in different cancer entities, including colorectal cancer (Borowczak et al., 2022).

We analysed all patient samples for the occurrence of *KRAS* mutations using mutant‐specific PCR to evaluate a possible stimulating effect of exercise on ctDNA concentrations. In our study, one patient was positive for the G12R *KRAS* mutation and had colon cancer with residual liver metastasis. Although *KRAS* is among the most commonly mutated oncogenes in numerous cancer types, *KRAS* mutations are present in only ∼11.6% of all carcinomas (Yang et al., 2023). The fraction of ctDNA, which is released by tumour cells, typically comprises ∼0.01%–5% of total cfDNA. However, depending on cancer type, stage, tumour burden and treatment status, the ctDNA fraction can be higher (Bronkhorst et al., 2019). In patients with colon cancer, Nakamura et al. (2021) identified a median *KRAS* mutant fraction of 0.20% (range 0.04%–68.99%). In our case, the fraction of ctDNA reduced from 2.162% to 0.369%. This can be attributed to the higher levels of background cfDNA, because the absolute ctDNA concentrations remained similar before and after the cycling test (0.106 vs. 0.14 ng/mL). The ctDNA and cfDNA concentrations at +90 min were similar to the Pre values. This finding highlights the importance of considering physical exercise as a preanalytical factor for cancer‐liquid biopsies. Kuligina et al. (2021) studied the effect of physical activity in a relevant real‐world scenario. In addition to the effects of circadian rhythm and food intake, they measured the concentration of ctDNA in 18 subjects 15 min before and 15 min after walking upstairs for two stairwells. They did not detect changes in tumour‐derived ctDNA copies. However, studies with varying relevant physical activity modes, times and loads should be performed to elucidate comprehensively the impact of exercise on ctDNA detection in different cancer types. Exercise could increase or decrease the levels of ctDNA in plasma samples, having advantages or disadvantages for ctDNA detection in subjects with undiagnosed disease (Neuberger & Simon, 2022).

Our study includes several limitations, which should be addressed in follow‐up studies with larger sample sizes and more homogeneous patient collectives. Initially, owing to the small sample size of the study, type II statistical errors are possible. Moreover, although we analysed the seven most common *KRAS* mutations, ctDNA was detected in only a single patient. Larger and well‐characterized cohorts need to be studied to identify the acute and chronic effects of exercise on ctDNA levels. Notably, during exercise, blood flow decreases in the abdominal organs, whereas the thoracic blood volume within the lung increases greatly (Flamm et al., 1990). It cannot be excluded that ctDNA increases during exercise in patients with lung cancer, because blood flow increases in this organ. In this case, physical activation could have beneficial effects on ctDNA detection. Additionally, we did not account for changes in plasma volume, which can occur after short‐duration, high‐intensity exercise. In elderly subjects with a healthy circulation, the plasma volume decreased by 14% after four progressive 5 min intervals on a cycle ergometer. In our case, the plasma volume changes could be responsible for the slight increase in ctDNA after exercise (Bjerre‐Bastos et al., 2022). The consideration of this bias is especially important for biomarkers that are regulated only slightly in response to exercise. Finally, although our control subjects were age matched and the number of steps during the test was similar, the healthy subjects performed higher maximal power output. It cannot be excluded that the higher load led to larger cfDNA increases, and future studies might include control subjects with similar cardiorespiratory fitness.

## 5 CONCLUSION

Here, we could show that cardiopulmonary exercise testing followed by cfDNA and cytokine analysis is easily feasible in cancer patients. The results of our pilot analysis hint at a different reaction to acute physical exercise by cancer patients compared with healthy control subjects with respect to cfDNA clearance by DNase I. In both cohorts, the cfDNA levels increase immediately after the exercise bout and reduce at least to Pre values by +90 min, whereas cfDNA and DNase I were correlated only in control subjects. The latter finding might indicate a disordered homeostasis in the cancer group, potentially involving other nucleases and mechanisms for cfDNA clearance than in healthy control subjects, which should be addressed in more detail in follow‐up studies. Exercise mainly leads to the release of neutrophil‐related cytokines, and further research is required to study the extent to which the beneficial health effects of exercise are related to neutrophil activation. Moreover, our study highlights the need to include physical activity in preanalytical considerations in cancer liquid biopsy.

## AUTHOR CONTRIBUTIONS

Tobias Ehlert, Suzan Botzenhardt, Elmo W. I. Neuberger, Alexandra Brahmer, Birgit Enders, Thomas Kindler, Achim Heintz, Carl C. Schimanski and Perikles Simon were responsible for the conception or design of the work and conducting the experiments. Tobias Ehlert, Suzan Botzenhardt, Alfonso De Falco, Patricia S. Hähnel, Thomas Kindler, Alexandra Brahmer, Perikles Simon and Elmo W. I. Neuberger were responsible for analysing and interpreting the data. All authors critically revised the work for important intellectual content. All authors have read and approved the manuscript and agree to be accountable for all aspects of the work in ensuring that questions related to the accuracy or integrity of any part of the work are appropriately investigated and resolved. All persons designated as authors qualify for authorship, and all those who qualify for authorship are listed.

## CONFLICT OF INTEREST

None declared.

## FUNDING INFORMATION

None.

## Data Availability

The data that support the findings of the present study are available from the corresponding author on reasonable request.
